# Changes in Sexual Function and Quality of Life After TVT Surgery in Women with Stress Urinary Incontinence: A Prospective Cohort Study

**DOI:** 10.3390/reports8030173

**Published:** 2025-09-07

**Authors:** Tamas Szabo, Melinda Ildiko Mitranovici, Janos Turos, Hilda Denes, Raluca Moraru, Lucian Puscasiu

**Affiliations:** 1 Doctoral School, University of Medicine, Pharmacy, Sciences and Technology “George Emil Palade”, 540142 Targu Mures, Romania; szabo_tamas91@yahoo.com (T.S.); jturos@yahoo.com (J.T.); deneshilda@yahoo.com (H.D.); puscasiu@gmail.com (L.P.); 2 Department of Obstetrics and Gynecology, Emergency County Hospital Hunedoara, 331057 Hunedoara, Romania; 3 Department of Anatomy, University of Medicine, Pharmacy, Sciences and Technology “George Emil Palade”, 540142 Targu Mures, Romania; raluca.moraru@umfst.ro

**Keywords:** stress urinary incontinence, quality of life, sexual function, surgical treatment, questionnaire

## Abstract

Involuntary urinary leakage due to stress urinary incontinence in women represents a widespread health condition that reduces quality of life. **Background**: Treatment with tension-free vaginal tape (TVT) remains the most used procedure, although its impact on quality of life, specifically regarding sexual function effects, has not been thoroughly investigated. The aim of our study is to achieve a broader understanding of the full range of outcomes after surgery, emotional well-being, and sexual function. **Materials and Methods**: The present prospective cohort study was conducted between 15 July 2023 and 15 June 2024 in the Emergency County Clinical Hospital Targu Mures, Department of Obstetrics and Gynecology. This is an investigation of TVT surgery and its impact on urinary incontinence, conducted by evaluating bladder dysfunction and sexual function before and after surgical intervention, as well as considering physical and psychological outcomes using specific questionnaires. **Results:** There was a 91.7% objective cure rate for incontinence, while urinary symptoms, sexual function, and emotional health significantly improved, urine leakage associated with strong urgency (*p* = 0.0002), urine leakage associated with coughing, sneezing, or laughing (*p* ≤ 0.0001), and patient sexual activity and emotional health also improved after surgery (*p* ≤ 0.0001). Furthermore, colorectal symptoms improved. **Conclusions**: This study emphasizes that for the best recovery of sexual and emotional health post-surgery, complete symptom removal is a requirement. Additionally, the significance of combined questionnaires in assessing treatment efficacy is highlighted. A larger sample size of patients and a longer follow-up are required before recommending this procedure as a standard treatment.

## 1. Introduction

Stress urinary incontinence (SUI) causes physical discomfort, emotional distress, social limitations, impaired daily functioning, and reduced sexual satisfaction [1,2]. These effects are important to women’s health because SUIs are common, particularly after childbirth. Emotional well-being, social engagement, and sexual function are also affected by urinary incontinence (UI), resulting in a reduction in the overall quality of life among women [3]. The physical discomfort compounds the psychological burden for a worsening life cycle.

Severe cases of SUI are treated with mid-urethral sling implantation, tension-free trans-obturator sub-urethral tapes, and single-incision slings [4,5,6]. Restoring continence has been shown to improve women’s bladders and, therefore, is key in these surgical interventions. Although SUI can be adequately treated surgically, there is insufficient evidence regarding the effect of this surgery on other aspects of women’s health [7,8].

There is a need to achieve a broader understanding of the full range of outcomes after surgery. This is possible by evaluating bladder dysfunction and sexual function before and after surgical intervention, as well as considering physical and psychological outcomes using specific questionnaires, such as the Pelvic Floor Impact Questionnaire, (PFIQ-7), Pelvic Floor Distress Inventory (PFDI-20), and Pelvic Organ Prolapse/Urinary Incontinence Sexual Function Questionnaire (PISQ-12), to evaluate the symptoms and their improvement after surgery [9,10,11,12].

The application of a mid-urethral sling is the gold standard in SUI [13]. The use of mini slings enhances sexual function. An important goal of SUI treatment is to improve quality of life [14]. The efficacy of mini slings is evaluated by transperineal ultrasonography and a self-reported questionnaire [15], but as was suggested, the effects of this surgical procedure are not evaluated properly.

The aim of our research is to evaluate the cure rates of TVT surgery, as well as the impact of this procedure on urinary incontinence symptoms, emotional well-being, and sexual function. Improvement in patient satisfaction was assessed using a combined questionnaire, which also evaluated the importance of specific questionnaires given pre- and post-operatively to the patients with SUI enrolled in our study.

## 2. Materials and Methods

The present prospective cohort study was conducted between 15 July 2023 and 15 June 2024 in the Emergency County Clinical Hospital Targu Mures, Department of Obstetrics and Gynecology. A total of 24 patients were included from a pool of 30 women diagnosed with pelvic floor disorders requiring surgical intervention who agreed to answer the questionnaire; 6 patients were lost to follow-up. The inclusion criteria consisted of confirmed SUI. Patients were excluded if they had a history of anti-incontinence surgery, pelvic organ prolapse, or other types of urinary incontinence. Also, patients with pathologies that generate incontinence were excluded, such as diabetes and neurological diseases. Informed consent was obtained from all subjects involved in this study. Our research was conducted in accordance with the Helsinki Declaration, with the approval of the Ethics Committee of Research UMFST Targu Mures for publication (No. 2433/10 July 2023).

Symptoms, daily activities, physical mobility, emotional well-being, and sexual function were assessed using the Pelvic Floor Impact Questionnaire (PFIQ-7), Pelvic Floor Distress Inventory (PFDI-20), and Pelvic Organ Prolapse/Urinary Incontinence Sexual Function Questionnaire (PISQ-12), a validated instrument that evaluates symptoms in patients with UI and/or POP [9,10,11,12,13,14]. The PFIQ-7 is a self-report questionnaire in which patients describe how their condition affects their quality of life and its impact on daily activities and emotional health. A 4-point Likert scale is used: 1 = “not at all”, 2 = “somewhat”, 3 = “moderately”, and 4 = “quite a bit”. A higher score indicates greater impairment of the person’s quality of life and daily activities.

The Pelvic Floor Distress Inventory (PFDI-20) is similar to PFIQ-7; it is a health-related questionnaire for women with pelvic floor disorders containing 20 questions. PFDI-20 includes the Urinary Distress Inventory-6 (UDI-6), Pelvic Organ Prolapse Distress Inventory-6 (POPDI-6), and the Colorectal–Anal Distress Inventory-8 (CRADI-8) and reflects the symptoms experienced by patients. A Likert scale similar to that used in PFIQ-7 is employed (possible values of 0 to 4). Higher scores indicate a greater impact on a patient’s life.

The Pelvic Organ Prolapse/Urinary Incontinence Sexual Function Questionnaire (PISQ-12) was used to assess the impact of urinary incontinence (UI) on sexual function in our patients before and after surgery. It is designed to evaluate sexual function in cases of pelvic floor disorder and evaluates three domains: behavioral effects, physical symptoms, and partner-related information regarding sexual activity. A 5-point Likert scale is used for each item; higher scores indicate better sexual function, where 0 represents “always” and 4 represents “never”. It was completed with the help of a doctor who knew the English language very well and translated the questions for the patients for self-completion by the patients.

The pre-operative examination included a 1 h pad test, urogynecological physical examination, and a self-administered questionnaire. Under general anesthesia, all patients underwent the retropubic sling procedure (Gynecare TVT blue, Ethicon Inc., Johnson & Johnson, Somerville, NJ, USA). The surgical procedure was performed according to the 1/3 rule, with tape tensioning achieved using an intraoperative cough test. The duration of hospitalization was between 2 and 3 days. No antibiotic administration was necessary. No intra- and post-operative complications were reported.

Follow-up visits were scheduled for 6 months after surgery and included a cough test, 1 h pad test, and pelvic floor ultrasound examination. Absorbent pad weight is measured, initially dried, then after one hour of use, it is re-weighed. All patients completed the PFDI-20, PFIQ-7, and PISQ-12 during the follow-up. The objective cure rate was defined as no leakage during the cough test with a bladder filling of approximately 300 mL and a negative 1 h pad test (≤2 g).

Descriptive statistical analysis was performed using PRISM 9 (Graphpad™ San Diego, CA, USA) and Microsoft Excel (Microsoft Office™). Results are reported as mean ± standard deviation. The statistical analysis included descriptive statistics (percentage and frequency) and elements of inferential statistics. To determine the association between qualitative variables, the chi-squared test was applied. The results were considered significant with a *p*-value < 0.05. We used the Graphpad Prism 9 software trial version to perform statistical analysis.

## 3. Results

The study included 24 participants who had a mean age of 53.1 ± 6.41 years. Employment status varied: 10 participants were employed, 10 were retired, and 4 were unemployed. Among the current or pre-existing medical problems reported by the participants, arterial hypertension was the most commonly mentioned (seven times). Other important comorbidities included thyroid disease (six cases) and cardiac disease (three cases). The most common complaint was urinary incontinence, which was reported by all 24 participants, with a symptom duration ranging from 6 months to 20 years (mean duration 4.98 ± 4.04 years) (Table 1).

**Figure 1:**
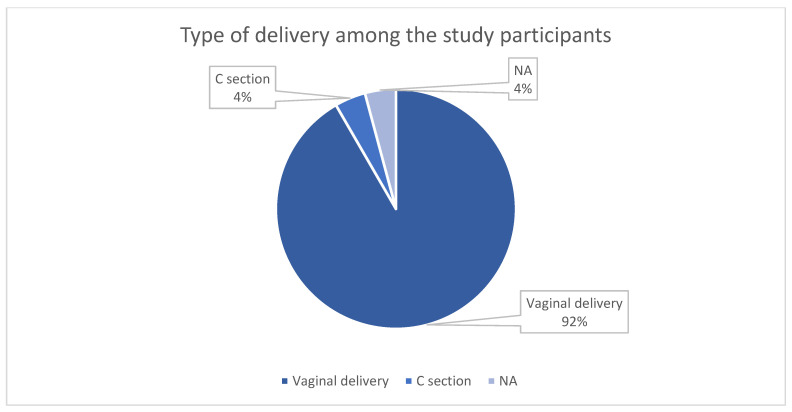
Types of deliveries.

The overwhelming majority of women had a history of vaginal delivery, which has an important impact on the pelvic floor. One participant had delivered via cesarean section, and another had no history of childbirth (Figure 1).

The study cohort had a mean of 2.92 ± 1.74 pregnancies, with a maximum of 9 pregnancies per participant. The mean number of deliveries was 1.83 ± 0.637. Ten women reported a history of abortion; the highest number of abortions for a single patient was seven (Table 2). The maximum newborn birth weight reported in this cohort was 4200 g, while the minimum birth weight was 2850 g, and the mean was 3542 ± 420 g. Regarding menstrual health, 17 participants reported no current menstrual cycles and 9 reported experiencing bleeding during menopause. All 24 women were sexually active at the time of examination (Table 2).

**Figure 2:**
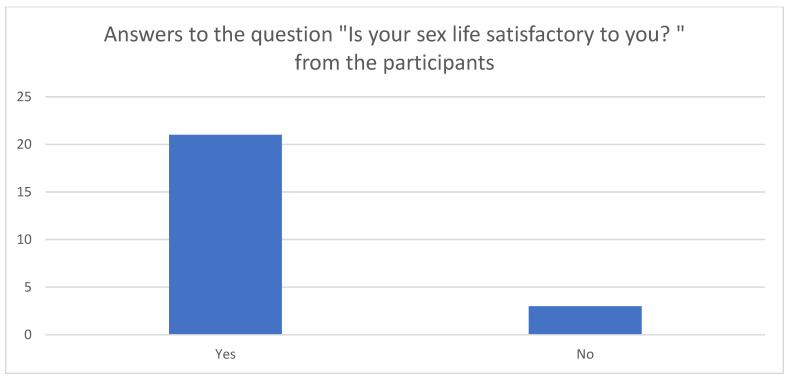
Satisfactory sexual life before surgery.

A total of 21 out of 24 women described their sexual life as being satisfactory, whereas 5 participants reported experiencing pain during intercourse (Figure 2). In terms of marital status, twenty-two participants were married, one was divorced, and one was not married.

A history of past or present smoking was identified in four women. Regular exercise was quoted only by three women, whilst alcohol consumption was reported in just one case. All patients denied any history of drug consumption. These aspects were not relevant for our research.

**Figure 3:**
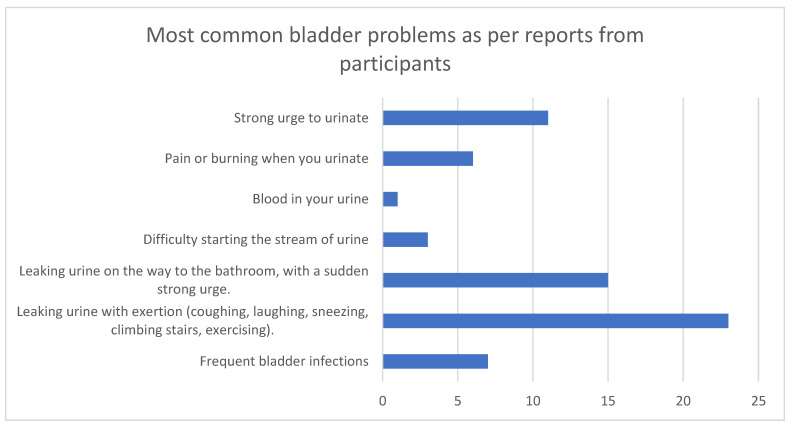
The most common bladder problems reported by patients.

The bladder problems most frequently reported by the participants were leaking urine with exertion (coughing, laughing, sneezing, climbing stairs, exercising) (mentioned 23 times), leaking urine on the way to the bathroom with a sudden strong urge (15 mentions), and a strong urge to urinate (Figure 3).

When asked how many times during the day they need to urinate, the mean study group value was 9.29 ± 5.75; the highest number reported was 30. On average, the women who participated in our study reported that they needed to wake up to urinate 2.50 ± 2.15 times per night. A total of 2 out of 24 women complained about having wet the bed before the date of the study, and 18 women reported using absorbent pads, which they replaced on average 4.06 ± 1.43 times per day. Just one patient complained of fecal incontinence among the 24 participants.

### The Utility of Questionnaires in Stress Urinary Incontinence Management

All patients completed the Pelvic Floor Impact Questionnaire (PFIQ-7), Pelvic Floor Distress Inventory (PFDI-20), and PISQ-12 urinary incontinence sexual questionnaire both before surgery and during post-operative follow-up.

Before surgery, the 24 participants stated they found household chores, such as cooking and cleaning, to be moderately to severely difficult as a result of bladder dysfunction. There is an association between surgery and bladder and urine symptoms. After the surgery, the majority of the subjects declared that the symptoms or conditions did not all affect their ability to do diverse physical activities, entertainment activities, travel by car away from home, and do social activities outside the home, and they also did not have any problems with emotional health (nervousness, depression, etc.) and feelings of frustration.

These data are presented in Table 3.

Regarding the ability to participate in diverse physical activities, there is a statistically significant difference between their experiences before and after surgery in terms of bladder or urine symptoms but not bowel and rectum or vaginal and pelvic symptoms (Table 4). We used the chi-squared test in all tables.

There were also statistically significant differences between their experiences before and after surgery regarding entertainment activities. Again, there was an association between surgery and bladder or urine symptoms; however, no significant association was observed in terms of bowel and rectum symptoms or vagina and pelvis pain (Table 5).

Surgery was associated with a statistically significant improvement in bladder and urinary symptoms in terms of the ability to travel by car away from home. However, bowel and rectum symptoms and vaginal and pelvic discomfort were not significantly ameliorated by surgery (Table 6).

Regarding social activities outside the home, surgery had a statistically significant impact on bladder and urinary symptoms. There was no significant impact of surgery on symptoms related to the bowel and rectum or vagina and pelvis (Table 7).

The surgical correction of bladder and urinary symptoms was associated with a statistically significant difference in emotional health (nervousness, depression, etc.). The same changes were not observed regarding bowel and rectum symptoms or vaginal and pelvic pain (Table 8).

The same effect was observed in terms of frustration: bladder and urinary symptom amelioration significantly improved patients’ feelings. (Table 9). Bowel-related issues had an impact on social and emotional well-being before and after surgery.

**Figure 4:**
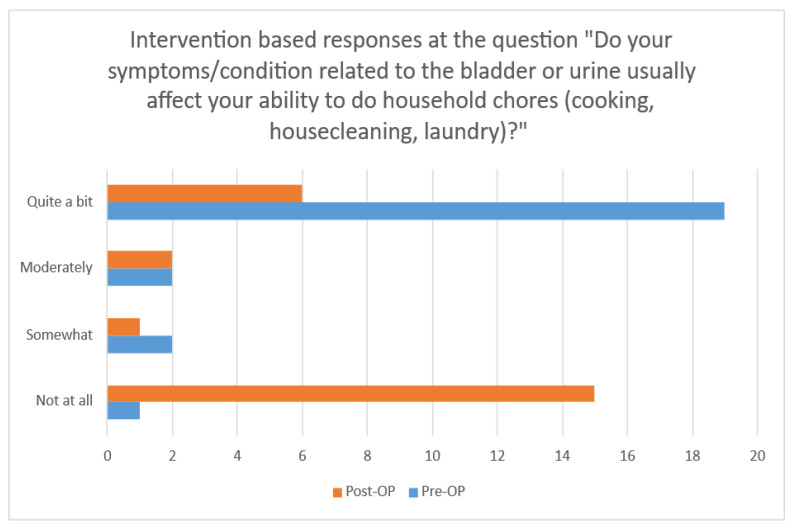
Association between household chores and SUI urinary symptoms before and after surgical intervention. Symptoms are significantly improved after surgery, and household chores are not affected after surgery.

The impact on urinary symptoms, which was impressively significant, is presented in Figure 4.

A 20-question questionnaire was used to assess the POP distress. The Pelvic Floor Distress Inventory (PFDI-20), which includes the Urinary Distress Inventory and the Colorectal Distress Inventory, is a valid tool to evaluate patient quality of life. Each question has five responses: not at all/somewhat/moderately/quite a bit/no (Table 10).

Before surgery, patients had a broad spectrum of distressing symptoms concerning pelvic organ prolapse. Regarding symptoms of pressure in the lower abdomen or the feeling of something falling in the patient’s vaginal area, the changes from before to after surgery are statistically significant (*p* = 0.0022). Regarding heaviness or dullness in the pelvic area, the procedure also reduced symptoms (*p* = 0.0232, respectively, *p* = 0.0367). The same effect was not observed for bowel movement, which was not significantly improved after surgery. While patients often complained of difficulty with bladder emptying before surgery, this decreased significantly after the surgical procedure (*p* = 0.0081). The changes from before to after surgery are not statistically significant regarding the need to push up with the fingers on a bulge in the vaginal area to complete urination, something that is more associated with pelvic organ prolapse than stress urinary incontinence.

There is no association between surgery and Colorectal–Anal Distress, except for those patients who experience a strong sense of urgency to have to rush to the bathroom while feeling a bowel movement (*p* = 0.0130) and those who complain of a bulge outside their bowel during a bowel movement (*p* = 0.0954).

While urinary distress problems are present before surgery, these symptoms have improved significantly post-operatively, regardless of whether we are talking about frequent urination (*p* ≤ 0.0001), urine leakage associated with strong urgency (*p* = 0.0002), urine leakage related to coughing or pressure in the abdomen (*p* ≤ 0.0001), or urine leakage in drops (*p* ≤ 0.0001). Additionally, patients who experienced difficulty in emptying their bladder and pain in the pelvic region or genital area before surgery reported a real improvement in their symptoms (*p* = 0.0067 and *p* = 0.0002).

PISQ-12 was used to evaluate the impact of SUI on sexual health in our patients before and after surgery. Three areas are considered: behavior, symptoms, and partner sexual impact. According to the PISQ-12 questionnaire, all three aspects assessed were improved by surgery, as shown in Table 11.

Many sexual problems were improved by this surgical procedure, including sexual desire (*p* ≤ 0.0001), having an orgasm (*p* = 0.0077), feeling sexually excited (*p* = 0.0026), being satisfied with a variety of sexual activities in their current sex life (*p* = 0.0006), and pain during sexual intercourse (*p* = 0.0011). None of our patients experienced urine leakage during sexual activity after surgery. Before surgery, the fear of incontinence (either stool or urine) restricted patient’s sexual activity; this aspect was significantly improved after surgery (*p* ≤ 0.0001). The same improvement was observed regarding avoiding sexual intercourse because of bulging in the vaginal area (*p* ≤ 0.0001).

No significant impact was observed regarding avoiding sexual intercourse because of negative emotional feelings, for example, guilt, fear, shame, or disgust, or related to partner sexual activity regarding a partner’s erection or premature ejaculation.

After surgery, the intensity of patients’ orgasms was significantly improved (*p* < 0.0001).

## 4. Discussion

Before surgery, the 24 participants stated that they found household chores, such as cooking and cleaning, to be moderately to severely difficult as a result of bladder dysfunction. There is an association between surgery, the pelvic floor, and bladder or urine symptoms. Regarding the ability to do household chores (cooking, housecleaning, laundry), the changes from before to after surgery are statistically significant regarding urinary leakage and other urinary symptoms. On the other hand, changes regarding the bowel and rectum are not significant. The PFIQ 7 questionnaire demonstrated its importance and seems to be a time-efficient method to assess symptoms and treatment effectiveness.

All patients complained of many symptoms concerning pelvic organ prolapse. The sensation of pressure in the lower abdomen significantly improved after surgery (*p* = 0.0022). In addition, vaginal bulging was reduced after surgery (*p* = 0.0232) and bladder emptying decreased (*p* = 0.0081). Bladder emptying was assessed by measuring residual urine on sonography. The same progress was not observed for bowel movement or colorectal distress. This may be because this procedure was being used for cases of stress urinary incontinence only. However, patients who had feelings of incomplete bowel emptying or who experienced bowel bulge before surgery declared an improvement after surgery (*p* = 0.0954). Moreover, patients who reported having uncontrolled stool leakage before the operation declared that this issue was solved by surgery (*p* = 0.0130).

Urinary distress symptoms were present before treatment but improved significantly after surgery. This was true for frequent urination (*p* ≤ 0.0001), urine leakage associated with strong urgency (*p* = 0.0002), and urine leakage associated with coughing, sneezing, or laughing (*p* ≤ 0.0001). The issue of drops of urine leakage was also improved by surgery (*p* ≤ 0.0001). A real improvement in symptoms with clinical relevance was obtained post-operatively. The Pelvic Floor Distress Inventory-20 was demonstrated by our research to be a valid tool for the evaluation of symptoms and treatment effects in women with pelvic floor disorders.

Sexual discomfort and sexual dysfunction were important issues because 12 participants complained of pain during intercourse before the surgical procedure and 14 feared incontinence would limit their sexual activity. All reports of pain during intercourse were post-operatively reduced after treatment (*p* = 0.0011). The participants reported that they experienced adverse emotional reactions, such as fear, shame, or guilt during intercourse, before surgery; however, these feelings were not significantly reduced after surgery. Furthermore, eight participants were concerned about vaginal bulging affecting sexual activity; this was significantly reduced post-operatively (*p* ≤ 0.0001).

In addition, the restriction of patient sexual activity due to fear of incontinence was significantly reduced after surgery (*p* ≤ 0.0001). Urine leakage during sexual activity was completely resolved by surgery. Regarding partner aspects, some of them did not improve, for example, erection problems or premature ejaculation, but these aspects are not related to surgery, while the orgasm intensity in the partner was significantly improved (*p* < 0.0001). The surgical procedure used in the present study has been proven as optimal for improving the sex lives of patients. The same observation was made by Abbas in their research [16].

The Pelvic Organ Prolapse/Urinary Incontinence Sexual Function Questionnaire (PISQ-12) is a valuable tool for clinicians to assess the impact of SUI on women’s sexual health and to evaluate the effectiveness of surgery, which in our case was aimed at improving the sexual function of our patients [17].

According to Melkie et al.’s study (2022), PFDI-20 and PFIQ-7, which were completed by 197 patients, are successful, reliable, and valid instruments to evaluate symptoms associated with pelvic organ prolapse [9]. The Spanish versions of the PISQ-12, PFIQ-7, and PFDI-20 questionnaires have shown good psychometric characteristics when used for the evaluation of patients with pelvic organ dysfunctions [10]. The same observation was made by Sanchez et al. in their study (2015). The responsiveness was higher for PFDI-20 than for PFIQ-7, meaning it is a valuable tool to assess the quality of life in patients with pelvic floor disorders. These tools effectively measure the efficacy of the treatment [11]. Colorectal–Anal Distress showed poor responsiveness according to Sanchez [11]. Our study emphasizes the importance of these questionnaires. PFDI-20 is recommended by the International Consultation on Incontinence (ICI) as the primary tool for assessing pelvic floor dyssynergy. Further studies are needed to evaluate these instruments [18].

The PFDI-20 and PISQ-12 questionnaires were given to 647 patients enrolled in Levis et al.’s study (2020); good results were obtained in the evaluation of pelvic floor disorder [19]. When used by 100 women with SUI, PFDI-20 and PFIQ-7 were found to be valid and reliable to evaluate patients complaining of PFDs [20]. The same observation was made by Arellano et al. (2024), who recommend that these tools be routinely administered in urogynecology units [21]. Good results were obtained from 155 women with urogenital issues who completed a PFDI-20 and Genital Self-Image Scale-20 (GSIS-20) questionnaire in Handelzalts et al.’s study (2017) [22]. Our study has shown the same importance of these tools in evaluating women with SUI.

Surgical treatment can significantly improve the quality of life of patients with SUI. In Guan’s review, through questionnaire evaluations, it was shown that colpocleisis may offer more advantages than transvaginal mesh-based repair (TVM) or other surgical procedures [23].

In Pauls et al.’s (2015) study, the PISQ-12 questionnaire was completed by 872 women with anal incontinence and seemed to be the most appropriate to characterize this condition [12].

According to Li (2025), TVT enhances sexual function for both patient and partner [24]. The same outcome was obtained in our study. In Van Isacker’s study (2025), coital urinary incontinence and worsened dyspareunia during intercourse were reported after sling placement [14].

The results of this study add to the growing literature examining the effects of pelvic floor disorder surgical interventions on women’s clinical and sexual function. TVT significantly improves quality of life (QoL) and sexual function in women with SUI [25,26,27]. We obtained similar results in our study. There was an improvement in urinary incontinence symptoms, physical exercise, and daily activity engagement both before and after the surgery. On the whole, there was a notable improvement in emotional well-being in this cohort, and the psychological and emotional distress that often accompanies PFDs, including social disengagement and anxiety, was alleviated. Furthermore, our findings regarding sexual function align with previous findings that TVT surgery improves sexual function in women [28,29,30]. In our study, we focused on improvements in sexual activity and satisfaction, decreases in fear of incontinence during sexual intercourse, and changes in adverse emotional reactions. Thus, surgical intervention allows for the resolution of physical symptoms and has a very beneficial effect on sexual health and intimacy, factors commonly ignored in the assessment of UI and POP treatments [31,32,33,34]. In addition, we used a combined questionnaire that refers to multiple aspects related to the SUI surgery. Although our participants’ QoL improved significantly and only a tiny percentage experienced complications, the high patient satisfaction rates observed in both TOT and TVT groups in their study mirror those obtained in our study.

A limitation of our study is that it includes only a small cohort of patients in a single-center study. The questionnaire did not include patient anamnestic data, so we cannot assess to what extent this information would help in risk stratification. The reproducibility and generalization are limited. Another limitation would be the lack of a control group and the short-term follow-up. In the absence of a control group, it is difficult to separate the impact of TVT from placebo or spontaneous change. Another limitation is that the questionnaires were not validated in the native language of the patients. However, our results align with those from several other studies, which is a strength of our research. Additionally, our study combined the questionnaires, which helped us to understand to what extent this procedure improves various aspects of quality of life and in what situations it proves ineffective, aspects that have clinical relevance. The combined questionnaire was necessary because each of these tools address different aspects: while PFIQ and PFDI-20 address symptoms, PISQ-12 addresses the impact of stress urinary incontinence on sexual function.

## 5. Conclusions

The findings of this study demonstrate that TVT surgery is a promising procedure that enhances both sexual function and quality of life in pelvic floor disorder patients. Moreover, our study emphasized the significance of combined questionnaires in assessing treatment efficacy. Prior research has proven how surgical treatments for SUI and POP provide substantial improvements across urinary incontinence symptoms, alongside improvements in sexual function and overall well-being. More studies evaluating the long-term psychological impacts and recovery effects of PFD treatment should be conducted to create a comprehensive PFD treatment strategy. A larger sample of patients and a longer follow-up are required before recommending this procedure as the standard treatment.

## Figures and Tables

**Table 1:** Demographic and clinical characteristics of the studied group.

Characteristic	Value
Number of Participants	24
Mean Age (years)	53.1 ± 6.41
**Employment Status**	
Employed	10
Retired	10
Unemployed	4
**Obstetric History**	
Mean Number of Pregnancies	2.92 ± 1.74
Mean Number of Deliveries	1.83 ± 0.637
History of Abortion (mean)	1.18 ± 1.56
**Comorbidities**	
Arterial Hypertension	7
Thyroid Disease	6
Cardiac Disease	3
**Sexual Activity**	
Sexual Activity at the Time of Study	24 (100%)
Mean Duration of Partner Commitment (years)	25.4 ± 9.31
**Lifestyle Factors**	
Smoking History	4
Regular Exercise	3
Alcohol Consumption	1
**Bladder Issues**	
Leaking Urine with Exertion	24 mentions
Leaking Urine with a Strong Urge	15 mentions
Strong Urge to Urinate	15 mentions
Frequency of Daytime Urination	9.29 ± 5.75 times/day
Frequency of Nocturia (Nighttime Urination)	2.50 ± 2.15 times/night
Use of Absorbent Pads	18 participants (4.06 ± 1.43 times/day)
History of Fecal Incontinence	1 participant

**Table 2:** Obstetric and menstrual history of participants.

Characteristic	Value
**Obstetric History**	
Mean Number of Pregnancies	2.92 ± 1.74
Maximum Number of Pregnancies	9
Mean Number of Deliveries	1.83 ± 0.637
Number of Women with a History of Abortion	10
Maximum Number of Abortions for a Single Participant	7
**Birth Weight**	
Maximum Birth Weight	4200 g
Minimum Birth Weight	2850 g
Mean Birth Weight	3542 ± 420 g
**Menstrual Health**	
Number of Women Reporting No Current Menstrual Cycles	17
Number of Women Reporting Bleeding During Menopause	9

**Table 3:** The association between household chores such as cooking and cleaning and bladder, bowel and rectum, or vaginal and pelvic symptoms.

Domain	Response	Pre-OP	Post-OP	*p*-Value
**Bladder and urine symptoms**	Not at all	1 (4.17%)	15 (62.50%)	0.0002
	Somewhat	2 (8.33%)	1 (4.17%)	
	Moderately	2 (8.33%)	2 (8.33%)	
	Quite a bit	19 (79.17%)	6 (25.00%)	
	Total	24 (100.00%)	24 (100.00%)	
**Bowel and rectum symptoms**	Not at all	17 (70.83%)	16 (66.67%)	0.6928
	Somewhat	1 (4.17%)	0 (0.00%)	
	Moderately	1 (4.17%)	2 (8.33%)	
	Quite a bit	5 (20.83%)	6 (25.00%)	
	Total	24 (100.00%)	24 (100.00%)	
**Vaginal and pelvic symptoms**	Not at all	17 (70.83%)	15 (62.50%)	0.9079
	Somewhat	1 (4.17%)	1 (4.17%)	
	Moderately	1 (4.17%)	2 (8.33%)	
	Quite a bit	5 (20.83%)	6 (25.00%)	
	Total	24 (100.00%)	24 (100.00%)	

**Table 4:** The ability to do diverse physical activities: bladder and urine symptoms, bowel and rectum symptoms, or pelvis and vagina symptoms.

Domain	Response	Pre-OP	Post-OP	*p*-Value
**Bladder and urine symptoms**	Not at all	1 (4.17%)	16 (66.67%)	<0.0001
	Somewhat	2 (8.33%)	0 (0.00%)	
	Moderately	5 (20.83%)	1 (4.17%)	
	Quite a bit	16 (66.67%)	7 (29.17%)	
	Total	24 (100.00%)	24 (100.00%)	
**Bowel and rectum symptoms**	Not at all	18 (75.00%)	16 (66.67%)	0.4842
	Somewhat	1 (4.17%)	0 (0.00%)	
	Moderately	0 (0.00%)	1 (4.17%)	
	Quite a bit	5 (20.83%)	7 (29.17%)	
	Total	24 (100.00%)	24 (100.00%)	
**Vaginal and pelvic symptoms**	Not at all	17 (70.83%)	16 (66.67%)	0.6928
	Somewhat	1 (4.17%)	0 (0.00%)	
	Moderately	1 (4.17%)	2 (8.33%)	
	Quite a bit	5 (20.83%)	6 (25.00%)	
	Total	24 (100.00%)	24 (100.00%)	

**Table 5:** Entertainment activities and the impact on bladder and urine, bowel and rectum, or pelvis and vagina symptoms.

Domain	Response	Pre-OP	Post-OP	*p*-Value
**Bladder and urine symptoms**	Not at all	4 (16.67%)	16 (66.67%)	0.0045
	Somewhat	2 (8.33%)	0 (0.00%)	
	Moderately	4 (16.67%)	2 (8.33%)	
	Quite a bit	14 (58.33%)	6 (25.00%)	
	Total	24 (100.00%)	24 (100.00%)	
**Bowel and rectum symptoms**	Not at all	18 (75.00%)	16 (66.67%)	0.2490
	Somewhat	1 (4.17%)	0 (0.00%)	
	Moderately	0 (0.00%)	3 (12.50%)	
	Quite a bit	5 (20.83%)	5 (20.83%)	
	Total	24 (100.00%)	24 (100.00%)	
**Vaginal and pelvic symptoms**	Not at all	18 (75.00%)	16 (66.67%)	0.2490
	Somewhat	1 (4.17%)	0 (0.00%)	
	Moderately	0 (0.00%)	3 (12.50%)	
	Quite a bit	5 (20.83%)	5 (20.83%)	
	Total	24 (100.00%)	24 (100.00%)	

**Table 6:** The ability to travel by car and the association with bladder and urinary, rectum and bowel, or pelvis and vagina symptoms.

Domain	Response	Pre-OP	Post-OP	*p*-Value
**Bladder and urinary symptoms**	Not at all	1 (4.17%)	14 (58.33%)	0.0004
	Somewhat	1 (4.17%)	2 (8.33%)	
	Moderately	7 (29.17%)	3 (12.50%)	
	Quite a bit	15 (62.50%)	5 (20.83%)	
	Total	24 (100.00%)	24 (100.00%)	
**Bowel and rectum symptoms**	Not at all	17 (70.83%)	16 (66.67%)	0.2487
	Somewhat	1 (4.17%)	0 (0.00%)	
	Moderately	0 (0.00%)	3 (12.50%)	
	Quite a bit	6 (25.00%)	5 (20.83%)	
	Total	24 (100.00%)	24 (100.00%)	
**Vaginal and pelvic symptoms**	Not at all	16 (66.67%)	16 (66.67%)	0.7212
	Somewhat	1 (4.17%)	0 (0.00%)	
	Moderately	1 (4.17%)	2 (8.33%)	
	Quite a bit	6 (25.00%)	6 (25.00%)	
	Total	24 (100.00%)	24 (100.00%)	

**Table 7:** Social activities in SUI and the impact on bladder and urine, bowel and rectum, and vaginal and pelvic symptoms.

Domain	Response	Pre-OP	Post-OP	*p*-Value
**Bladder and urine symptoms**	Not at all	2 (8.33%)	14 (58.33%)	0.0034
	Somewhat	5 (20.83%)	2 (8.33%)	
	Moderately	3 (12.50%)	2 (8.33%)	
	Quite a bit	14 (58.33%)	6 (25.00%)	
	Total	24 (100.00%)	24 (100.00%)	
**Bowel and rectum symptoms**	Not at all	17 (70.83%)	15 (62.50%)	0.9279
	Somewhat	1 (4.17%)	1 (4.17%)	
	Moderately	1 (4.17%)	1 (4.17%)	
	Quite a bit	5 (20.83%)	7 (29.17%)	
	Total	24 (100.00%)	24 (100.00%)	
**Vaginal and pelvic symptoms**	Not at all	17 (70.83%)	15 (62.50%)	0.9279
	Somewhat	1 (4.17%)	1 (4.17%)	
	Moderately	1 (4.17%)	1 (4.17%)	
	Quite a bit	5 (20.83%)	7 (29.17%)	
	Total	24 (100.00%)	24 (100.00%)	

**Table 8:** Emotional health in SUI and bladder and urinary symptoms, bowel and rectum symptoms, and pelvis and vagina symptoms.

Domain	Response	Pre-OP	Post-OP	*p*-Value
**Bladder and urinary symptoms**	Not at all	4 (16.67%)	17 (70.83%)	0.0005
	Somewhat	2 (8.33%)	3 (12.50%)	
	Moderately	3 (12.50%)	0 (0.00%)	
	Quite a bit	15 (62.50%)	4 (16.67%)	
	Total	24 (100.00%)	24 (100.00%)	
**Bowel and rectum symptoms**	Not at all	18 (75.00%)	18 (75.00%)	0.6077
	Somewhat	1 (4.17%)	2 (8.33%)	
	Moderately	0 (0.00%)	1 (4.17%)	
	Quite a bit	5 (20.83%)	3 (12.50%)	
	Total	24 (100.00%)	24 (100.00%)	
**Vaginal and pelvic symptoms**	Not at all	18 (75.00%)	18 (75.00%)	0.6077
	Somewhat	1 (4.17%)	2 (8.33%)	
	Moderately	0 (0.00%)	1 (4.17%)	
	Quite a bit	5 (20.83%)	3 (12.50%)	
	Total	24 (100.00%)	24 (100.00%)	

**Table 9:** Patients’ feelings regarding SUI and bladder and urinary symptoms, bowel and rectum symptoms, and pelvis and vagina symptoms.

Domain	Response	Pre-OP	Post-OP	*p*-Value
**Bladder and urinary symptoms**	Not at all	2 (8.33%)	21 (87.50%)	<0.0001
	Somewhat	6 (25.00%)	2 (8.33%)	
	Moderately	4 (16.67%)	0 (0.00%)	
	Quite a bit	12 (50.00%)	1 (4.17%)	
	Total	24 (100.00%)	24 (100.00%)	
**Bowel and rectum symptoms**	Not at all	18 (75.00%)	21 (87.50%)	0.2728
	Somewhat	1 (4.17%)	2 (8.33%)	
	Moderately	2 (8.33%)	1 (4.17%)	
	Quite a bit	3 (12.50%)	0 (0.00%)	
	Total	24 (100.00%)	24 (100.00%)	
**Vaginal and pelvic symptoms**	Not at all	18 (75.00%)	21 (87.50%)	0.2728
	Somewhat	1 (4.17%)	2 (8.33%)	
	Moderately	2 (8.33%)	1 (4.17%)	
	Quite a bit	3 (12.50%)	0 (0.00%)	
	Total	24 (100.00%)	24 (100.00%)	

**Table 10:** Pelvic Floor Distress Inventory (PFDI-20).

Question	Response	Pre-Op (%)	Post-Op (%)	*p*-Value
**Q1: Pressure in the pelvis**	Not at all	0.00%	16.67%	0.0022
	Somewhat	41.67%	16.67%	
	Moderately	25.00%	0.00%	
	Quite a bit	8.33%	4.17%	
	No	25.00%	62.50%	
**Q2: Dullness or heaviness in the pelvic area**	Not at all	8.33%	12.50%	0.0232
	Somewhat	41.67%	16.67%	
	Moderately	12.50%	0.00%	
	Quite a bit	12.50%	4.17%	
	No	25.00%	66.67%	
**Q3: Bulge or something falling out in the patient’s vaginal area**	Not at all	12.50%	12.50%	0.0367
	Somewhat	29.17%	4.17%	
	Moderately	8.33%	0.00%	
	Quite a bit	0.00%	0.00%	
	No	50.00%	83.33%	
**Q4: Have to push on the vagina or the rectum to have a bowel movement**	Not at all	0.00%	4.17%	0.2674
	Somewhat	16.67%	4.17%	
	Moderately	4.17%	0.00%	
	Quite a bit	4.17%	0.00%	
	No	75.00%	91.67%	
**Q5: Feeling of incomplete bladder emptying?**	Not at all	0.00%	8.33%	0.0081
	Somewhat	37.50%	16.67%	
	Moderately	20.83%	8.33%	
	Quite a bit	16.67%	0.00%	
	No	25.00%	66.67%	
**Q6: Need to push up on a bulge in the vaginal area to complete urination**	Not at all	0.00%	4.17%	0.2351
	Somewhat	16.67%	4.17%	
	Moderately	0.00%	0.00%	
	Quite a bit	0.00%	0.00%	
	No	83.33%	91.67%	
**Colorectal–Anal Distress**				
**Q7: Need to strain to have a bowel movement**	Not at all	4.17%	4.17%	0.1322
	Somewhat	12.50%	0.00%	
	Moderately	8.33%	0.00%	
	No	75.00%	95.83%	
**Q8: Not completely emptying your bowels after a bowel movement**	Not at all	4.17%	16.67%	0.1076
	Somewhat	33.33%	8.33%	
	Moderately	8.33%	4.17%	
	No	50.00%	70.83%	
**Q9: Lose solid stool beyond your control**	Not at all	0.00%	4.17%	0.0945
	Somewhat	16.67%	0.00%	
	Moderately	4.17%	0.00%	
	No	79.17%	95.83%	
**Q10: Lose liquid stool beyond your control**	Not at all	0.00%	0.00%	0.2805
	Somewhat	8.33%	4.17%	
	Moderately	8.33%	0.00%	
	No	83.33%	95.83%	
**Q11: Lose gas from the rectum beyond your control**	Not at all	0.00%	4.17%	0.2215
	Somewhat	25.00%	12.50%	
	Moderately	8.33%	0.00%	
	No	62.50%	83.33%	
**Q12: Passing stool associated with pain**	Not at all	0.00%	4.17%	0.3557
	Somewhat	20.83%	8.33%	
	Moderately	4.17%	0.00%	
	Quite a bit	0.00%	4.17%	
	No	75.00%	83.33%	
**Q13: Urge to rush to the bathroom for bowel movement**	Not at all	0.00%	8.33%	0.0130
	Somewhat	29.17%	0.00%	
	Moderately	16.67%	8.33%	
	No	50.00%	83.33%	
**Q14: Bowel bulges out during a bowel movement**	Not at all	0.00%	0.00%	0.0954
	Somewhat	16.67%	0.00%	
	Moderately	4.17%	0.00%	
	Quite a bit	0.00%	4.17%	
	No	79.17%	95.83%	
**Urinary Distress**				
**Q15: Frequent urination**	Not at all	0.00%	4.17%	<0.0001
	Somewhat	0.00%	20.83%	
	Moderately	33.33%	4.17%	
	Quite a bit	45.83%	4.17%	
	No	20.83%	66.67%	
**Q16: Urgency with urine leakage**	Not at all	0.00%	4.17%	0.0002
	Somewhat	29.17%	20.83%	
	Moderately	20.83%	4.17%	
	Quite a bit	41.67%	4.17%	
	No	8.33%	66.67%	
**Q17: Leakage when coughing, sneezing, or laughing**	Not at all	4.17%	0.00%	<0.0001
	Somewhat	16.67%	4.17%	
	Moderately	33.33%	0.00%	
	Quite a bit	37.50%	4.17%	
	No	8.33%	91.67%	
**Q18: Urine leakage, small drops**	Not at all	0.00%	4.17%	<0.0001
	Somewhat	8.33%	20.83%	
	Moderately	45.83%	0.00%	
	Quite a bit	45.83%	4.17%	
	No	0.00%	70.83%	
**Q19: Difficulty emptying bladder**	Not at all	4.17%	8.33%	0.0067
	Somewhat	29.17%	25.00%	
	Moderately	16.67%	4.17%	
	Quite a bit	29.17%	0.00%	
	No	20.83%	62.50%	
**Q20: Pain/discomfort in lower abdomen or genital area**	Not at all	12.50%	4.17%	0.0002
	Somewhat	20.83%	8.33%	
	Moderately	29.17%	0.00%	
	Quite a bit	20.83%	4.17%	
	No	16.67%	83.33%	

**Table 11:** PISQ-12—Pelvic Organ Prolapse/Urinary Incontinence Sexual Function Questionnaire.

Question	Response	Pre-OP	Post-OP	*p*-Value
**1. Feeling sexual desire**	Never	1 (4.17%)	1 (4.17%)	<0.0001
	Rarely	19 (79.17%)	3 (12.50%)	
	Sometimes	2 (8.33%)	7 (29.17%)	
	Most of the time	2 (8.33%)	13 (54.17%)	
	Always	0 (0.00%)	0 (0.00%)	
	Total	24 (100.00%)	24 (100.00%)	
**2. Orgasm during sexual intercourse**	Never	1 (4.17%)	1 (4.17%)	0.0077
	Rarely	12 (50.00%)	1 (4.17%)	
	Sometimes	4 (16.67%)	6 (25.00%)	
	Most of the time	3 (12.50%)	10 (41.67%)	
	Always	4 (16.67%)	6 (25.00%)	
	Total	24 (100.00%)	24 (100.00%)	
**3. Sexual excitement when having sexual activity**	Never	1 (4.17%)	0 (0.00%)	0.0026
	Rarely	9 (37.50%)	0 (0.00%)	
	Sometimes	7 (29.17%)	7 (29.17%)	
	Most of the time	2 (8.33%)	11 (45.83%)	
	Always	5 (20.83%)	6 (25.00%)	
	Total	24 (100.00%)	24 (100.00%)	
**4. Satisfaction with a variety of sexual activities**	Never	3 (12.50%)	0 (0.00%)	0.0006
	Rarely	10 (41.67%)	1 (4.17%)	
	Sometimes	5 (20.83%)	4 (16.67%)	
	Most of the time	2 (8.33%)	14 (58.33%)	
	Always	4 (16.67%)	5 (20.83%)	
	Total	24 (100.00%)	24 (100.00%)	
**5. Pain during sexual intercourse**	Never	7 (29.17%)	10 (41.67%)	0.0011
	Rarely	2 (8.33%)	12 (50.00%)	
	Sometimes	8 (33.33%)	2 (8.33%)	
	Most of the time	5 (20.83%)	0 (0.00%)	
	Always	2 (8.33%)	0 (0.00%)	
	Total	24 (100.00%)	24 (100.00%)	
**6. Leak urine during sexual activity**	Never	5 (20.83%)	24 (100.00%)	
	Rarely	2 (8.33%)	0 (0.00%)	
	Sometimes	10 (41.67%)	0 (0.00%)	
	Most of the time	5 (20.83%)	0 (0.00%)	
	Always	2 (8.33%)	0 (0.00%)	
	Total	24 (100.00%)	24 (100.00%)	
**7. Fear of stool or urine incontinence during sexual activity**	Never	4 (16.67%)	20 (83.33%)	<0.0001
	Rarely	1 (4.17%)	3 (12.50%)	
	Sometimes	9 (37.50%)	1 (4.17%)	
	Most of the time	8 (33.33%)	0 (0.00%)	
	Always	2 (8.33%)	0 (0.00%)	
	Total	24 (100.00%)	24 (100.00%)	
**8. Avoiding sexual intercourse because of bulging in vagina**	Never	4 (16.67%)	22 (91.67%)	<0.0001
	Rarely	7 (29.17%)	1 (4.17%)	
	Sometimes	7 (29.17%)	1 (4.17%)	
	Most of the time	5 (20.83%)	0 (0.00%)	
	Always	1 (4.17%)	0 (0.00%)	
	Total	24 (100.00%)	24 (100.00%)	
**9. Negative emotional reactions during intercourse (fear, disgust, shame, or guilt)**	Never	8 (33.33%)	21 (87.50%)	
	Rarely	3 (12.50%)	3 (12.50%)	
	Sometimes	6 (25.00%)	0 (0.00%)	
	Most of the time	6 (25.00%)	0 (0.00%)	
	Always	1 (4.17%)	0 (0.00%)	
	Total	24 (100.00%)	24 (100.00%)	
**10. Partner erection problems that affect sexual activity?**	Never	20 (83.33%)	21 (87.50%)	0.8939
	Rarely	3 (12.50%)	2 (8.33%)	
	Sometimes	1 (4.17%)	1 (4.17%)	
	Most of the time	0 (0.00%)	0 (0.00%)	
	Always	0 (0.00%)	0 (0.00%)	
	Total	24 (100.00%)	24 (100.00%)	
**11. Partner premature ejaculation that affects sexual activity?**	Never	19 (79.17%)	20 (83.33%)	0.5988
	Rarely	4 (16.67%)	4 (16.67%)	
	Sometimes	1 (4.17%)	0 (0.00%)	
	Most of the time	0 (0.00%)	0 (0.00%)	
	Always	0 (0.00%)	0 (0.00%)	
	Total	24 (100.00%)	24 (100.00%)	
**12. Intensity of the orgasms compared with the past**	Much less intense	13 (54.17%)	0 (0.00%)	<0.0001
	Less intense	9 (37.50%)	5 (20.83%)	
	Same intensity	2 (8.33%)	4 (16.67%)	
	More intense	0 (0.00%)	9 (37.50%)	
	Much more intense	0 (0.00%)	6 (25.00%)	
	Total	24 (100.00%)	24 (100.00%)	

## Data Availability

Data are available at the Department of Obstetrics and Gynecology of the University of Medicine, Pharmacy, Sciences and Technology “George Emil Palade”.

## Abbreviations

The following abbreviations are used in this manuscript:

TVT	Tension-free vaginal tape
SUI	Stress urinary incontinence
UI	Urinary incontinence
PFIQ-7	Pelvic Floor Impact Questionnaire
PFDI-20	Pelvic Floor Distress Inventory
PISQ-12	Pelvic Organ Prolapse/Urinary Incontinence Sexual Function Urinary Distress
UDI-6	Inventory-6
POPDI-6	Pelvic Organ Prolapse Distress Inventory-6
CRADI-8	Colorectal–Anal Distress Inventory-8
